# A thiol-based intramolecular redox switch in four-repeat tau controls fibril assembly and disassembly

**DOI:** 10.1016/j.jbc.2021.101021

**Published:** 2021-07-31

**Authors:** Hilary A. Weismiller, Tyler J. Holub, Brad J. Krzesinski, Martin Margittai

**Affiliations:** Department of Chemistry and Biochemistry, University of Denver, Denver, Colorado, USA

**Keywords:** aggregation, Alzheimer's disease, amyloid, conformation, disulfide, fibril, prion, redox switch, seeding barrier, tau protein, thiol, 3R, three-repeat tau, 4R, four-repeat tau, AD, Alzheimer's disease, BCA, bicinchoninic acid, CW, continuous wave, EPR, electron paramagnetic resonance, GdnHCl, guanidine hydrochloride, H_2_O_2_, hydrogen peroxide, MTSL, [1-oxyl-2,2,5,5-tetramethyl-Δ3-pyrroline-3-methyl]methanethiosulfonate, Ox, oxidized, Red, reduced, TCEP, Tris(2-carboxyethyl)phosphine, TEM, transmission electron microscopy, ThT, thioflavin T

## Abstract

Oxidative stress has been implicated in the pathogenesis and progression of several tauopathies, including Alzheimer's disease. The deposition of fibrillar inclusions made of tau protein is one of the pathological hallmarks of these disorders. Although it is becoming increasingly evident that the specific fibril structure may vary from one tauopathy to another and it is recognized that different types of isoforms (three-repeat and four-repeat tau) can be selectively deposited, little is known about the role oxidation may play in aggregation. Four-repeat tau contains two cysteines that can form an intramolecular disulfide bond, resulting in a structurally restrained compact monomer. There is discrepancy as to whether this monomer can aggregate or not. Using isolated four-repeat tau monomers (htau40) with intramolecular disulfide bonds, we demonstrate that these proteins form fibrils. The fibrils are less stable than fibrils formed under reducing conditions but are highly effective in seeding oxidized tau monomers. Conversely, a strong seeding barrier prevents incorporation of reduced tau monomers, tau mimics in which the cysteines have been replaced by alanines or serines, and three-repeat tau (htau23), a single-cysteine isoform. The barrier also holds true when seed and monomer types are reversed, indicating that oxidized and reduced tau are incompatible with each other. Surprisingly, fibrils composed of compact tau disaggregate upon reduction, highlighting the importance of the intramolecular disulfide bond for fibril stability. The findings uncover a novel binary redox switch that controls the aggregation and disaggregation of these fibrils and extend the conformational spectrum of tau aggregates.

Fibrils composed of the microtubule-associated protein tau are a defining pathological feature of Alzheimer's disease (AD) and various other neurodegenerative disorders, collectively known as tauopathies (1, 2, 3). There is increasing evidence that short fibrils and small oligomers of tau can transfer from one neuron to another resulting in the recruitment of endogenous tau monomers into these aggregates and the spreading of tau pathology throughout the brain (4, 5). Similar to prions that are linked to fatal neurodegenerative disorders such as Creutzfeldt–Jakob disease, bovine spongiform encephalopathy, and scrapie (6), tau protein can replicate its conformation through template-assisted conversion (7). In this process, intrinsically disordered tau monomers assume the same parallel in-register *β* structure as the fibril, once recruited onto its ends. The fibril thus serves as a seed or a nucleus that bypasses the thermodynamically unfavorable early nucleation events, thereby accelerating fibril formation (8). Repeated cycles of fibril breakage and elongation are implicated in the propagation of the original fibril conformer (9). The biological mechanisms that mediate tau fibril breakage are still poorly understood although molecular chaperones of the Hsp70 system might be involved (10).

There are six different tau isoforms expressed in the adult human brain that vary by the presence or the absence of the second of four C-terminally located microtubule-binding repeats and the inclusion of zero, one, or two inserts in the protein's N-terminal region (11). The microtubule-binding repeats in tau play a special role because they become part of the fibril core, whereas the flanking regions form a largely disordered fuzzy coat (12, 13). Based on the number of microtubule-binding repeats, tau isoforms can be grouped into three-repeat (3R) tau and four-repeat (4R) tau. The largest tau isoform, htau40 or 2N4R, contains 441 amino acids; the smallest tau isoform, htau23 or 0N3R, contains 352 amino acids. In AD, all six tau isoforms are deposited as fibrils. In other tauopathies, including corticobasal degeneration and Pick's disease, there is a preferential deposition of only 4R tau or 3R tau, respectively. This deposition behavior can be explained by sequence-based structural incompatibilities between monomers and seeds (14). Recent cryo-EM studies have revealed that the conformations of the fibrils in these diseases are unique and that each tauopathy may be characterized by a single fold (15). In contrast, fibrils that are formed *in vitro*, particularly those that are composed of 4R tau, are mainly ensembles of different conformers (16, 17, 18, 19).

Although it is not clear what factors define the prevailing structure of tau fibrils, it appears that fibril-associating cofactors (20, 21, 22), tau mutations (23), and post-translational modifications (24) among others could play a significant role. One of the early tau modifications that had been postulated in tau nucleation is the formation of disulfide bonds (25). These bonds may form when reactive oxygen species in dysfunctional mitochondria of aging neurons cause an imbalance in cellular redox homeostasis (26). 3R tau contains a single cysteine at position 322 in the third repeat. 4R tau contains two cysteines: one at position 322 and the other one at position 291 in the second repeat. 3R tau can only form intermolecular disulfide bonds, whereas 4R tau can form both intermolecular and intramolecular disulfide bonds. There is strong evidence that intermolecular disulfide bonds between two tau monomers facilitate tau aggregation (27, 28, 29). However, these bonds have not yet been identified in any of the cryo-EM structures of disease-derived tau fibrils (30, 31, 32, 33). The formation of intramolecular disulfide bonds in 4R tau generates compact monomers that are structurally constrained (25, 34). Since both cysteines are employed in this bond, the proteins are unable to form disulfide linkages with other tau molecules. There has been some discrepancy in the literature as to whether compact tau monomers are aggregation resistant (34) or not (35). Previous experiments that demonstrated fibril formation of compact tau monomers did not separate these monomers from disulfide-linked dimers (35); and it was suggested that the presence of tau dimers may have facilitated aggregation (34). The latter study separated the monomers but used a truncated variant of tau (K18) lacking residues that may be part of the fibril core (30). Therefore, it is not clear whether some of the aggregation resistance of compact tau monomers could have been attributed to the shortened amino acid sequence of K18.

Here, we set out to clarify the aggregation properties of isolated compact monomers of full-length tau (htau40). The study shows that these monomers are aggregation competent, forming fibrils that selectively recruit other compact tau monomers, but not tau monomers lacking the intramolecular disulfide bond. A large fraction of the tau fibrils disaggregates when subjected to reducing conditions, revealing a novel molecular switch that is sensitive to changes in redox potential.

## Results

### Htau40 monomers with intramolecular disulfide bonds can be efficiently isolated

**Figure 1:**
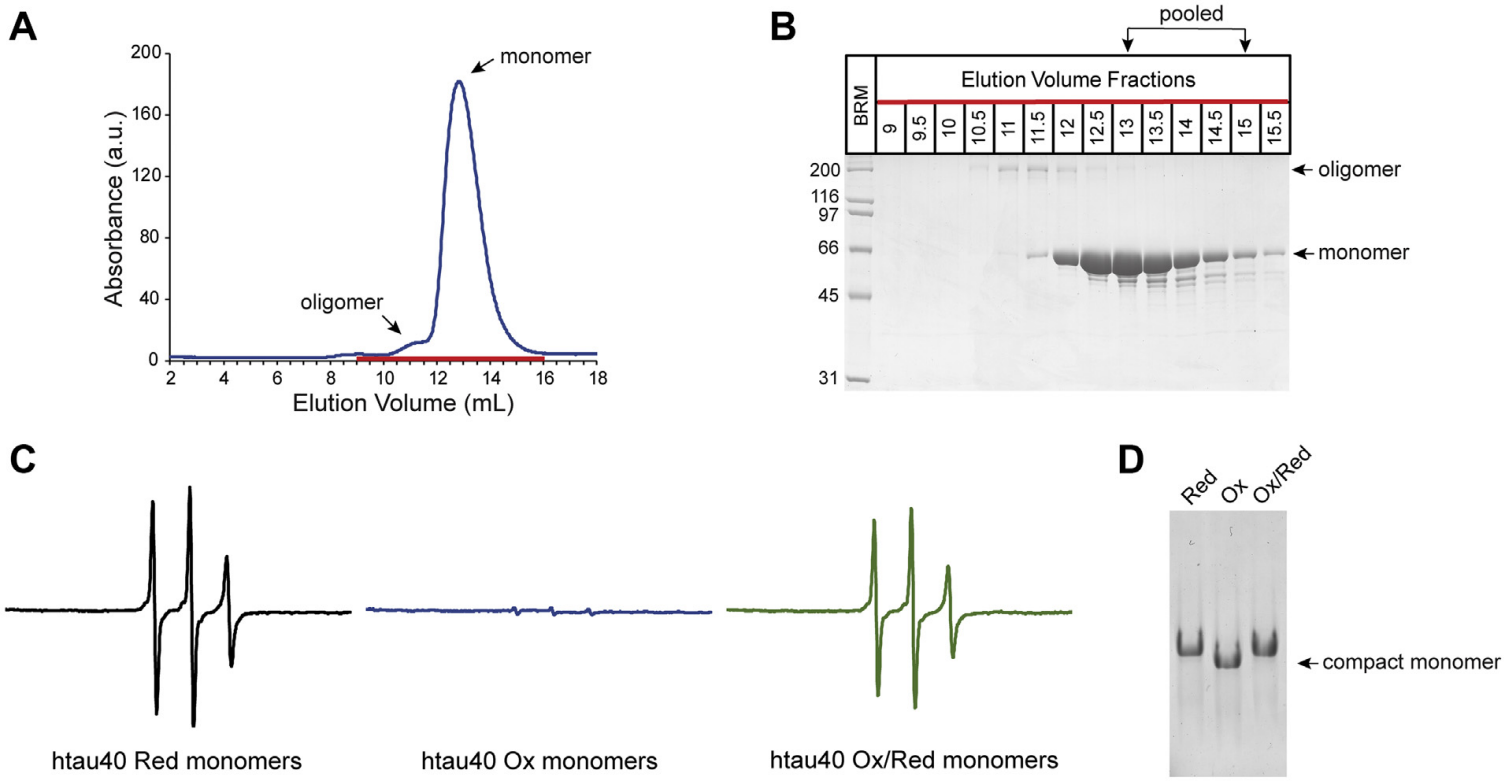
**Htau40 monomers with intramolecular disulfide bonds are isolated by size-exclusion chromatography.***A*, purified htau40 monomers (20 μM) were oxidized for 15 h at 22 °C with hydrogen peroxide (5 mM) and then subjected to size-exclusion chromatography (Superdex 200). *B*, fractions corresponding to the peak region in the chromatogram were analyzed by nonreducing SDS-PAGE and Coomassie staining. *C*, pooled fractions of oxidized htau40 monomers were reacted with a 10-fold molar excess of the thiol-reactive label, MTSL, and then analyzed by CW EPR (center spectrum). Equivalent analysis was performed for reactions involving reduced htau40 monomers (*left spectrum*) and oxidized htau40 monomers that were reduced with DTT prior to labeling (*right spectrum*). The concentration of tau protein in the EPR measurements was 5 μM. Scan width = 150 G. Same axes are used for all three plots. *D*, native PAGE of reduced monomers (Red), oxidized monomers (Ox), and oxidized monomers after reaction with DTT (Ox/Red). BRM, broad range marker; CW, continuous wave; EPR, electron paramagnetic resonance; MTSL, [1-oxyl-2,2,5,5-tetramethyl-Δ3-pyrroline-3-methyl]methanethiosulfonate.

In a first step, we set out to separate compact tau monomers from higher molecular weight species. To achieve this, purified htau40 monomers were incubated with hydrogen peroxide (H_2_O_2_) to promote disulfide bond formation and then subjected to size-exclusion chromatography to separate tau species with intramolecular disulfide bonds from those with intermolecular disulfide bonds (Fig. 1*A*). The collected fractions were analyzed by SDS-PAGE in the absence of reducing agent to ensure that all disulfide bonds remained intact. Fractions containing monomeric species could be clearly differentiated from those with higher order species and were pooled for further experiments (Fig. 1*B*).

The presence of an intramolecular disulfide bond in the monomeric species was verified by cross-linking experiments using the paramagnetic nitroxide label, [1-oxyl-2,2,5,5-tetramethyl-Δ3-pyrroline-3-methyl]methanethiosulfonate (MTSL) (36). This label selectively reacts with thiols but not disulfide groups. When oxidized htau40 monomers were subjected to MTSL labeling, the continuous wave (CW) electron paramagnetic resonance (EPR) spectrum showed only negligible signal (Fig. 1*C*, *center spectrum*), suggesting that free cysteines were not present in these monomers. In contrast, when reduced htau40 monomers were subjected to the same labeling procedure or when oxidized htau40 monomers were reacted with reducing agent (DTT) prior to labeling, EPR spectra with similar sharp lines were observed (Fig. 1*C*, *left* and *right spectra*). These spectra indicate that the tau monomers were intrinsically disordered (7, 37) and that the disulfide bonds in oxidized tau were reduced back to thiols. An overoxidation to sulfinic or sulfonic acid can be excluded because these oxidation states of sulfur cannot be reduced by DTT (38).

To independently confirm the existence of the intramolecular disulfide bond in htau40, we next subjected oxidized and reduced monomers to native gel electrophoresis. Oxidized monomers migrated faster through the gel than reduced monomers (Fig. 1*D*), consistent with a more compact structure (39). Importantly, when oxidized monomers were treated with DTT prior to electrophoresis, the proteins migrated indistinguishably from reduced monomers (Fig. 1*D*), suggesting that the intramolecular disulfide bond had been converted back to thiols and that the proteins had lost their compact shape.

The combined data indicate that the protocol for isolating tau monomers with intramolecular disulfide bonds was effective; these monomers will henceforth be referred to as htau40 oxidized (Ox).

### Htau40 oxidized monomer forms fibrils capable of self-seeding

**Figure 2:**
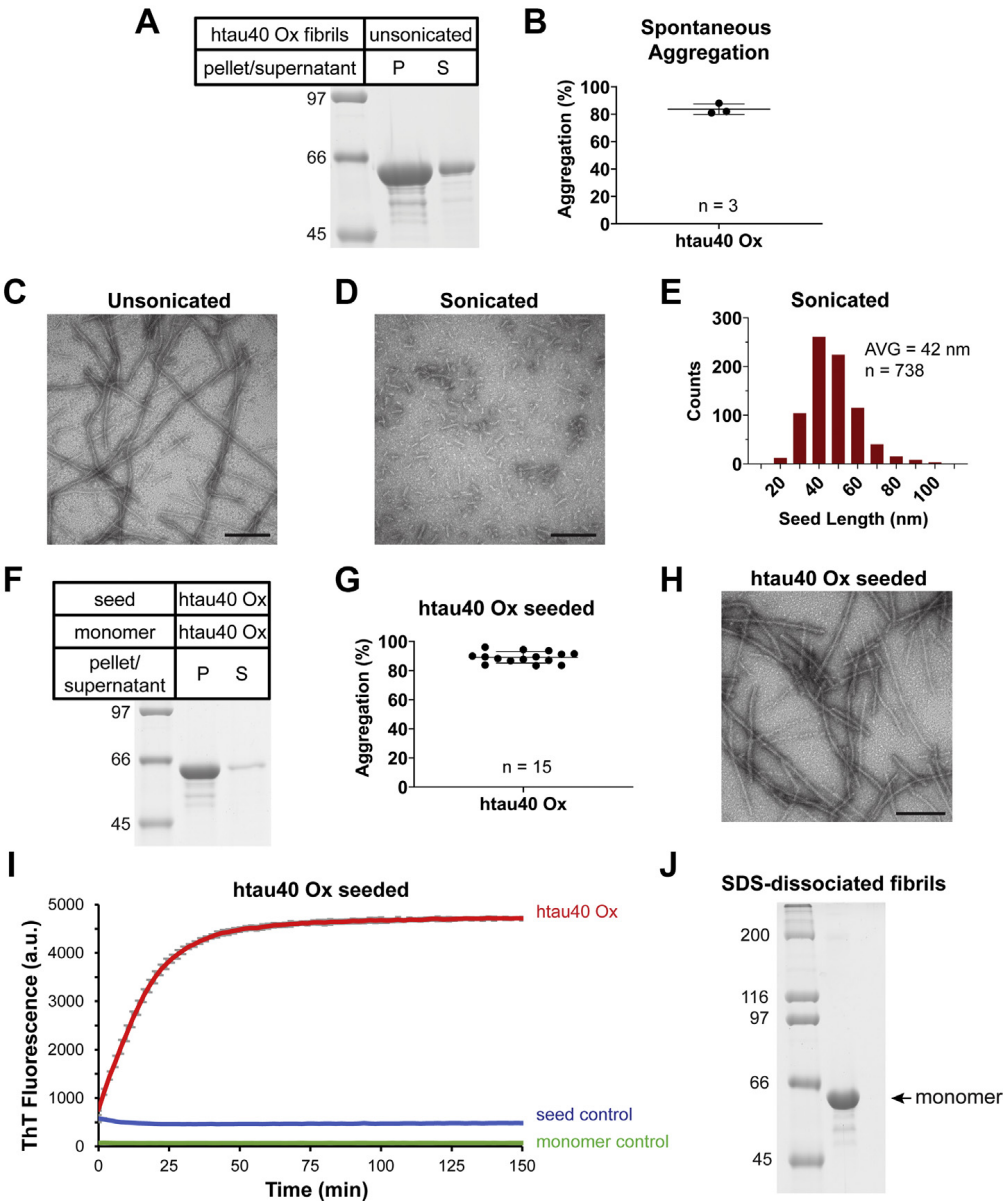
**Spontaneously nucleated fibrils of htau40 Ox seed homotypic growth.***A*, to test for spontaneous aggregation, 25 μM htau40 Ox monomers were combined with 50 μM heparin and incubated under agitation for 5 to 7 days at 37 °C. Upon centrifugation, equivalent volumes of pellet and supernatant were subjected to SDS-PAGE and visualized by Coomassie staining. *B*, quantitative analysis by densitometry depicted as a scatter plot. Shown are values for three independent measurements with bars for mean and standard deviation. *C* and *D*, negative-staining EM images of htau40 Ox fibrils taken before (*C*) and after (*D*) sonication. The scale bar represents 100 nm. *E*, size distribution of sonicated fibrils. *F* and *G*, to assess homotypic growth, 10 μM htau40 Ox monomer was mixed with 10% htau40 Ox seed (monomer equivalents) and 20 μM heparin, incubated quiescently at 37 °C for 20 to 24 h and then sedimented. The samples were analyzed by SDS-PAGE (*F*) and gel densitometry (*G*) as described in *A* and *B*. *H*, negative-staining EM image of fibrils produced by seeding. The scale bar represents 100 nm. *I*, ThT fluorescence measurements monitoring growth of htau40 Ox monomers onto htau40 Ox seeds (*red trace*) (10 μM monomers, 10% seeds). ThT measurements of only monomers (*green trace*) or only seeds (*blue trace*) served as controls. All measurements were performed in triplicate. Error bars represent means ± SD. *J*, to verify that the intramolecular disulfide bonds stayed intact during aggregation and did not reshuffle with neighboring tau molecules forming intermolecular disulfide bonds, homotypically seeded htau40 Ox fibrils were dissociated and analyzed by SDS-PAGE under nonreducing conditions. AVG, average length; n, number of independent replicates; Ox, oxidized; P, pellet; S, supernatant; ThT, thioflavin T.

To test the ability of isolated htau40 Ox monomers to spontaneously form fibrils, the monomers were first combined with heparin, a cofactor known to facilitate tau aggregation (40), and then incubated under agitating conditions for 5 to 7 days at 37 °C. Analysis by SDS-PAGE revealed that after sedimentation, most of the tau protein (84%) was enriched in the pellet (Fig. 2, *A* and *B*), suggesting that the protein had assembled into aggregates. To characterize the morphological properties of these aggregates, the samples were imaged by negative-staining transmission electron microscopy (TEM). All aggregates were of fibrillar nature; amorphous structures were noticeably absent (Fig. 2*C*). Shearing of the fibrils by tip sonication resulted in shorter fibrillar stubs, or seeds, with an average length of 42 nm (Fig. 2, *D* and *E*). When these seeds were offered to a pool of oxidized monomers and incubated quiescently for 20 to 24 h, tau protein was mainly found in the pellet (89%) following sedimentation (Fig. 2, *F* and *G*), indicating that oxidized tau monomers had been recruited onto oxidized seeds. Importantly, htau40 Ox monomers that were incubated for the same amount of time, but in the absence of seeds, did not aggregate (Fig. S1), demonstrating that the fibrils were not the result of spontaneous nucleation. The fibrillar nature of the assembled structures was confirmed by TEM (Fig. 2*H*). Seeded growth of htau40 Ox monomers onto htau40 Ox seeds was also observed using thioflavin T (ThT), a dye that exhibits increased fluorescence intensity upon binding to amyloid fibrils (Fig. 2*I*, *red trace*). As expected, no growth was observed in the absence of seeds as evidenced by the unaltered ThT fluorescence over time (Fig. 2*I*, *green trace*). In addition, there was no change in ThT fluorescence when seeds were incubated in the absence of tau monomers (Fig. 2*I*, *blue trace*) suggesting that the seeds remained intact.

When the fibrils from seeded reactions were dissociated by SDS and analyzed on a nonreducing gel, tau was exclusively monomeric (Fig. 2*J*), indicating that the intramolecular disulfide bond in tau did not reshuffle with neighboring tau molecules in the fibril. Such reshuffling would have resulted in higher molecular weight species in the gel. Together, the results suggest that htau40 Ox monomers form fibrils and that these fibrils are capable of efficient self-seeding.

### Cross-seeding barrier prevents recruitment of noncompact monomers onto htau40 Ox seeds

When tau monomers bind onto the ends of the fibril, they assume the same shape, perpetuating the original conformation of the seed as identical residues of incoming molecules stack on top of each other. Differences in sequence between monomer and seed can hinder the recruitment, in some cases abrogating it altogether (14, 41, 42). Post-translational modifications could have similar effects.

**Figure 3:**
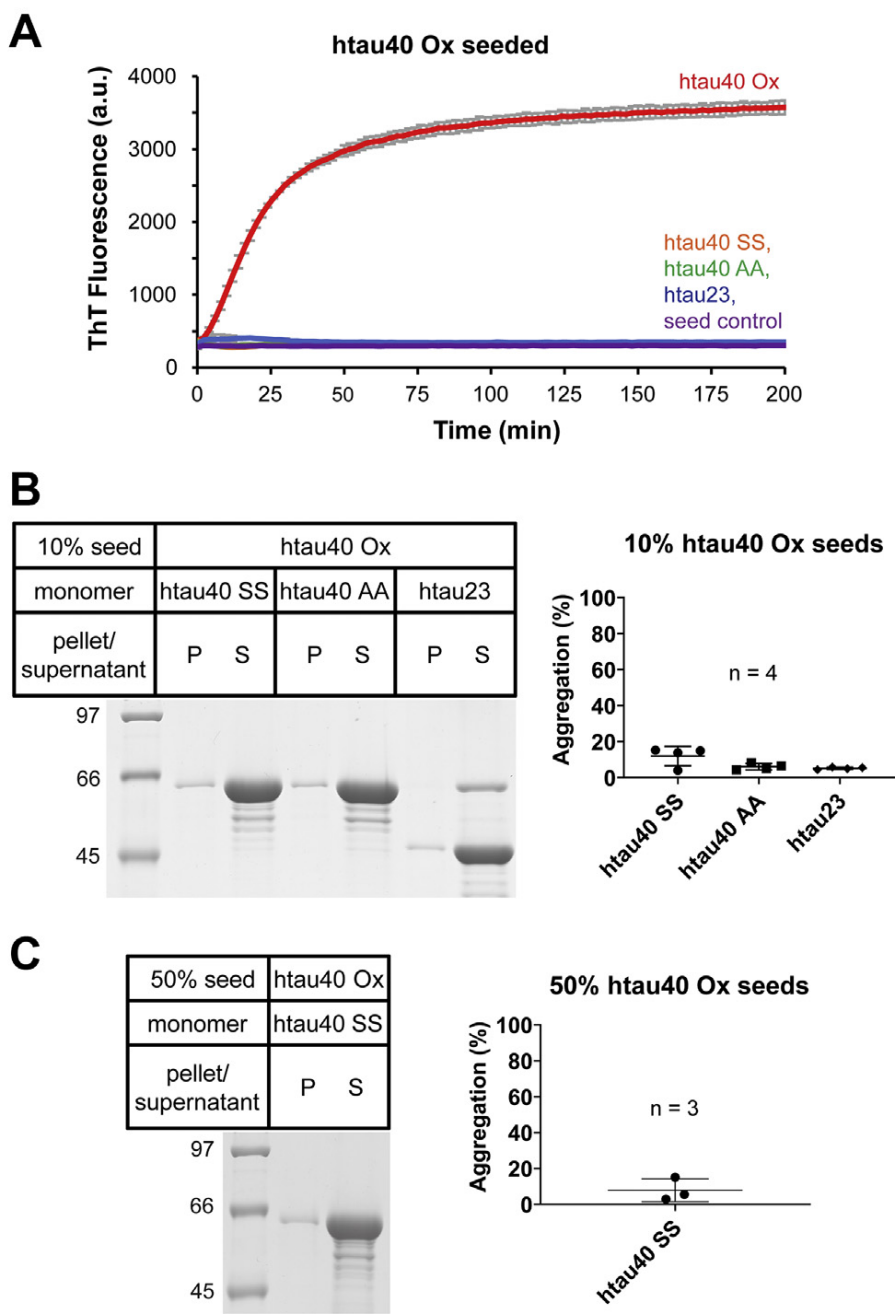
**Tau monomers require an intramolecular disulfide bond to grow onto htau40 Ox seeds.***A*, seeding capacity of htau40 Ox seeds monitored by ThT fluorescence. Ten percent seeds of htau40 Ox were mixed with 10 μM tau monomers (different variants), and ThT fluorescence was measured over time at 37 °C. Homotypic growth with htau40 Ox monomers (*red trace*), heterotypic growth with htau40 SS, htau40 AA, or htau23 monomers (*orange*, *green*, and *blue traces*, respectively). Htau40 Ox seed control without monomers added (*purple trace*). All reactions were performed in triplicate. *B*, monomer incorporation into seeds was separately examined by SDS-PAGE and Coomassie staining (*left panel*). After incubation of 10% seeds with 10 μM monomers for 5 days at 37 °C, the samples were centrifuged at 130,000*g*. Equivalent volumes of pellets and supernatants were loaded onto a gel. Quantitative analysis of seeded growth was performed using gel densitometry (*right panel*). *C*, monomer incorporation at increased seed concentration (50%) was analyzed by SDS-PAGE (*left panel*) and quantified by densitometry (*right panel*). All error bars represent means ± SD. n, number of independent replicates; Ox, oxidized; P, pellet; S, supernatant; ThT, thioflavin T.

In a next set of experiments, we sought to determine whether tau monomers that lack intramolecular disulfide bonds can be recruited onto htau40 Ox seeds. The monomers chosen to probe this question were two different variants of htau40 in which the native cysteines were replaced with serines (htau40 SS) or alanines (htau40 AA), and htau23, the shortest tau isoform, containing only a single cysteine at position 322. When these monomers were combined with 10% htau40 Ox seeds, no increase in ThT fluorescence was observed (Fig. 3*A*), indicating that the proteins were not recruited onto the fibrils. Sedimentation experiments in which the samples were centrifuged for 30 min at 130,000*g* after prolonged incubation confirmed these results. Only negligible amounts of protein were observed in the fibril pellets (Fig. 3*B*). Under the given sedimentation procedure, unreacted seeds, like monomers, remain in the supernatant, because of their small molecular size (43). To determine whether there is a subpopulation of oxidized seeds that can recruit variant tau, the seed concentration was increased to 50% (molar monomer equivalents) and offered htau40 SS monomers. Even then, recruitment of tau monomers was negligible, as unreacted seeds and monomers remained in the supernatant (Fig. 3*C*). This suggests that only monomers with an intramolecular disulfide bond can be recruited onto htau40 Ox seeds. To exclude the possibility that htau40 Ox monomers are recruited because of modifications other than the disulfide bond, we next treated htau40 AA and htau40 SS monomers with H_2_O_2_ and then offered them to htau40 Ox seeds. Again, the monomers did not incorporate (Fig. S2), corroborating the critical role of the disulfide bond for recruitment.

**Figure 4:**
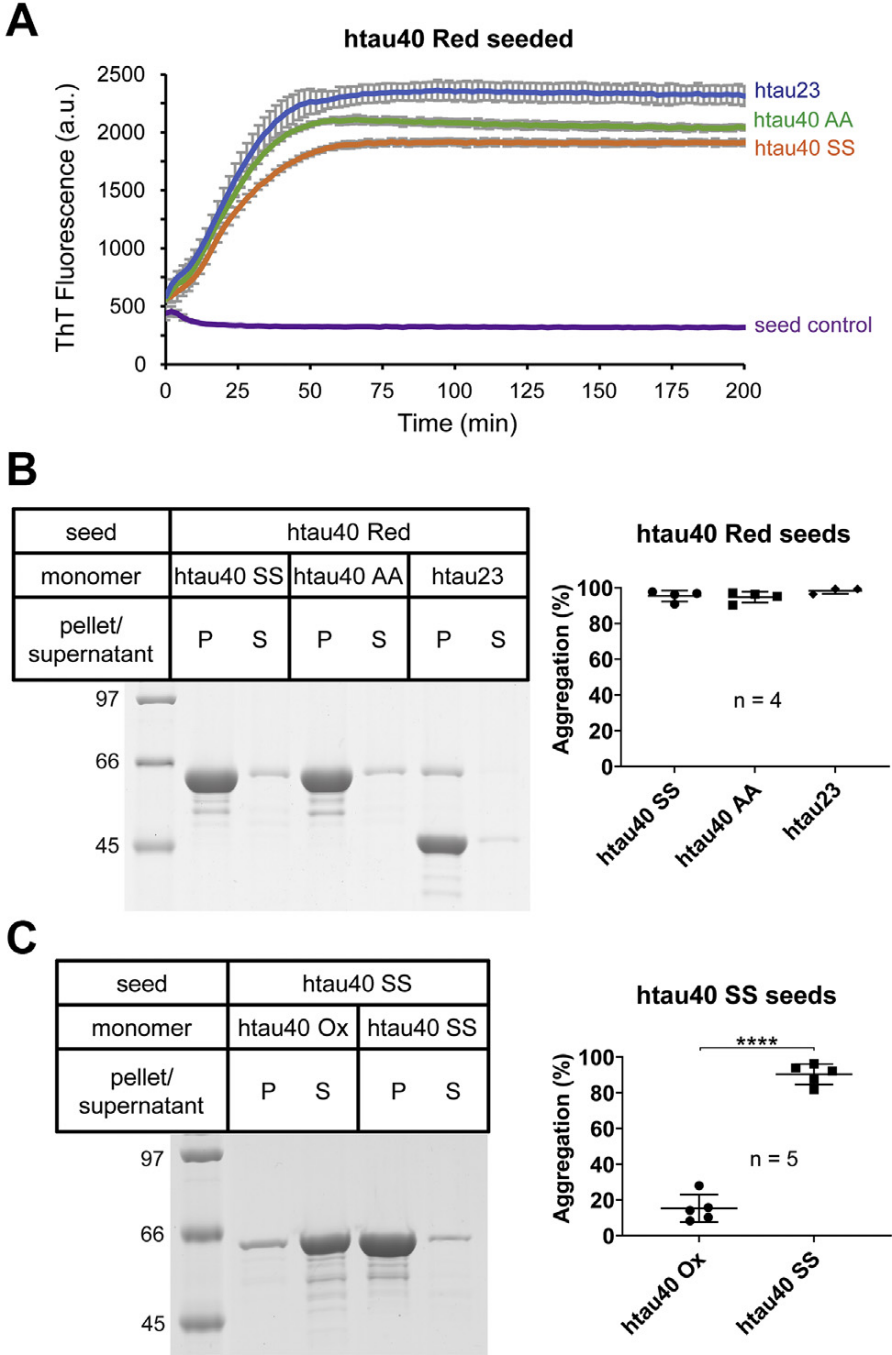
**Tau monomers without intramolecular disulfide bond are recruited onto nonoxidized htau40 seeds.***A*, 10% htau40 Red seeds were mixed with 10 μM variant tau monomers that lack intramolecular disulfide bonds and incubated at 37 °C. ThT fluorescence was measured over time. Monomers added: htau23 (*blue trace*), htau40 AA (*green trace*), htau40 SS (*orange trace*), and seed control (*purple trace*). All reactions were performed in triplicate. *B*, the reactions were repeated in the absence of ThT, incubated for 20 to 24 h, and then sedimented at 130,000*g*. SDS-PAGE analysis (*left panel*). Densitometric quantification (*right panel*). *C*, htau40 SS seeds were incubated with monomers of htau40 Ox and htau40 SS and sedimented as before. SDS-PAGE (*left panel*). Densitometric quantification (*right panel*). Error bars represent mean ± SD. Paired *t* test for statistical comparison: ∗∗∗∗*p* < 0.0001. n, number of replicates; Ox, oxidized; P, pellet; Red, reduced; S, supernatant; ThT, thioflavin T.

Finally, to rule out the possibility that untreated monomeric tau variants (no H_2_O_2_) were somehow damaged during purification, we combined them with htau40 wildtype fibrils formed in the presence of a reducing agent (referred to as htau40 reduced (Red) fibrils). These fibrils do not contain compact monomers. All three tau variants (htau40 SS, htau40 AA, and htau23) effectively grew onto the seeds (Fig. 4, *A* and *B*), confirming that the variants were functionally intact. Notably, reversing the seeding reactions, that is, offering htau40 Ox monomers to htau40 SS seeds failed to produce any fibrils (Fig. 4*C*). Combined, the data suggest that cross-seeding barriers prevent recruitment of nondisulfide-bonded monomers onto htau40 Ox seeds and *vice versa*.

### Reduced htau40 monomers are not recruited onto htau40 Ox seeds

**Figure 5:**
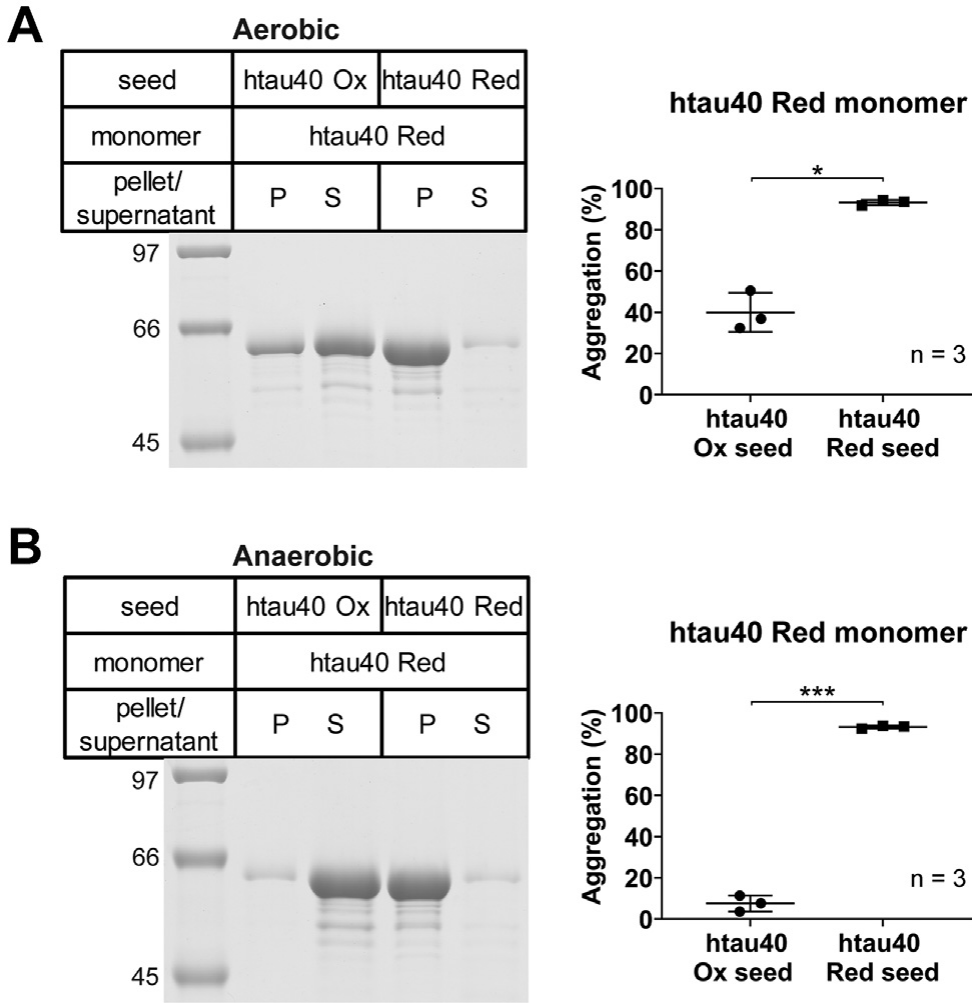
**The oxidation status of htau40 monomers determines whether they are recruited onto htau40 Ox seeds.***A*, Red htau40 monomers were combined with either htau40 Ox or htau40 Red seeds and incubated for 20 to 24 h at 22 °C under aerobic conditions. After centrifugation, the samples were analyzed by SDS-PAGE and Coomassie staining (*left panel*) and quantified by densitometry (*right panel*). *B*, the incubations were repeated at 37 °C under anaerobic conditions and analyzed as in *A*. Statistical comparison was performed using paired *t* tests. ∗*p* < 0.013 and ∗∗∗*p* < 0.0006. n, number of independent replicates; Ox, oxidized; P, pellet; Red, reduced; S, supernatant.

The htau40 SS and htau40 AA variants used in the previous experiments were mimics of wildtype htau40 in the reduced form, unable to form disulfide bonds. The substitutions of the cysteines, however, also altered the amino acid sequence, raising the question whether the growth incompatibilities could have been attributed to the inability of stacking nonidentical amino acids (alanines and serines onto cysteines) rather than the difference in structure between seed and substrate (compact *versus* noncompact). To distinguish between these two effects, we next examined the recruitment of wildtype htau40 (containing cysteines) onto htau40 Ox seeds. The experiments were performed under both, aerobic and anaerobic conditions. Under aerobic conditions, htau40 monomers are expected to slowly convert into the disulfide-linked form because of the presence of dissolved oxygen. Under anaerobic conditions, such conversion does not occur. All monomers were taken up under anaerobic conditions to suppress premature oxidation. These monomers (10 μM) were then mixed with 10% htau40 Ox seeds and quiescently incubated at 22 °C on the bench with lids kept open (aerobic) or at 37 °C in the anaerobic chamber with lids kept closed. After 22 to 24 h, the samples were sedimented and analyzed by SDS-PAGE. Under aerobic conditions, a significant fraction (40%) of the protein was observed in the pellet (Fig. 5*A*), suggesting that some of the monomers were recruited onto the seeds. Under anaerobic conditions, only a small fraction (8%) was in the pellet (Fig. 5*B*), indicating that in this case recruitment was greatly inhibited. These results were specific to htau40 Ox seeds, as parallel reactions with htau40 Red seeds resulted in effective growth (93%) under both aerobic and anaerobic conditions (Fig. 5). Collectively, the results suggest that htau40 monomers are only recruited onto htau40 Ox seeds after formation of an intramolecular disulfide bond. Reduced htau40 monomers that lack such bond are not recruited and thus behave similar as the tau SS and AA variants.

Remarkably, htau40 monomers in which the two cysteines were crosslinked with bis-maleimidoethane (creating an 8 Å spacer arm between them) were not recruited either (Fig. S3), suggesting that the cysteines not only need to be crosslinked but also need to be properly spaced.

### Htau40 fibrils formed in the presence of Cu^2+^ exhibit the same seeding properties as fibrils formed from H_2_O_2_-oxidized tau

In a next set of experiments we examined whether the unique seeding properties of htau40 Ox fibrils could also be observed when instead of H_2_O_2_, Cu^2+^ was used as an oxidizing agent. There is evidence that at least part of the oxidative stress observed in AD might be linked to copper dishomeostasis (44). Previous studies have used Cu^2+^ as an oxidizing agent for tau (34) and demonstrated the preferential formation of an intramolecular disulfide bridge (45).

**Figure 6:**
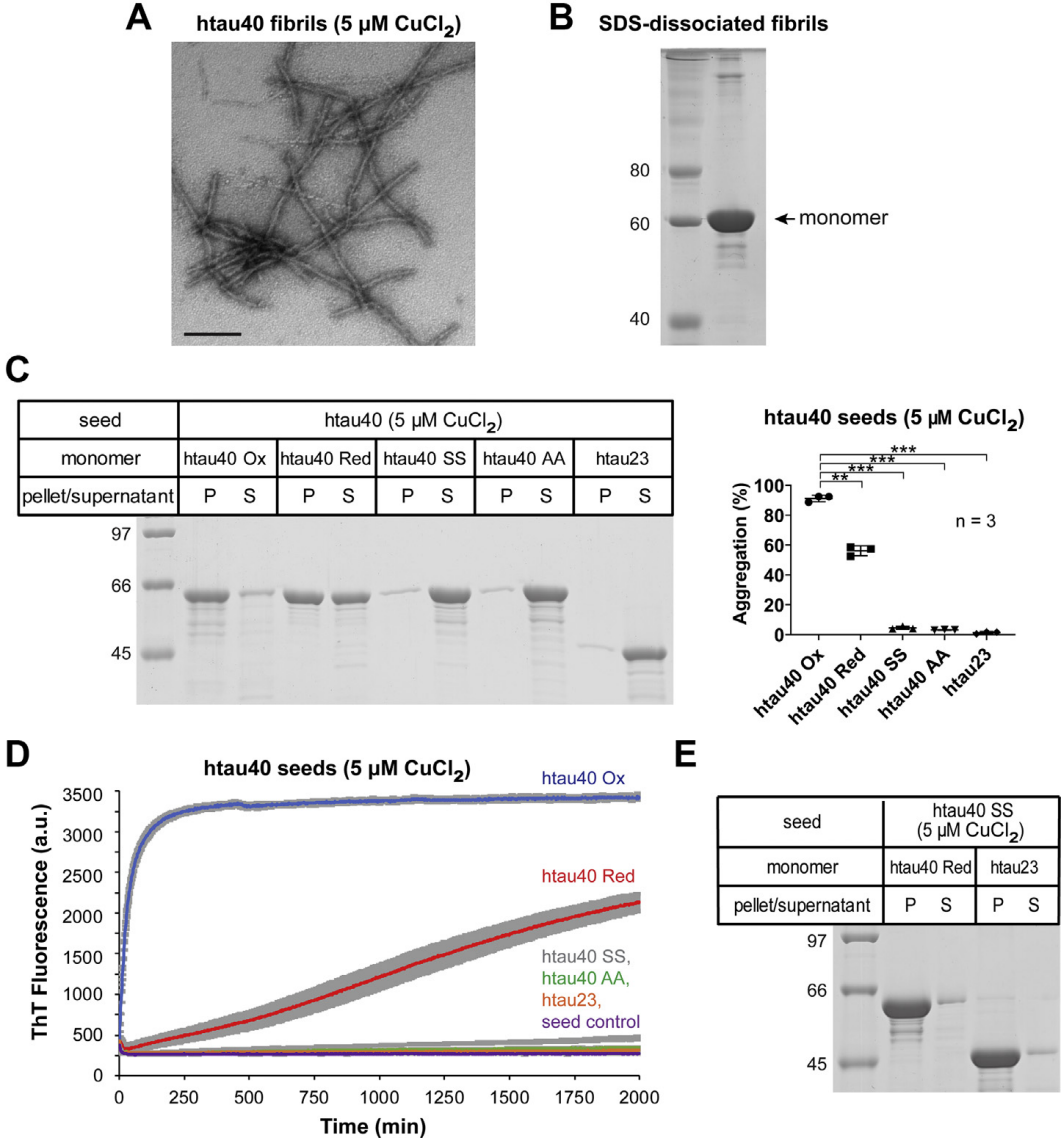
**The seeding properties of htau40 fibrils formed in the presence of copper (II) match those of fibrils formed from H**_**2**_**O**_**2**_**-oxidized htau40.** To form fibrils, 25 μM htau40 monomers were mixed with 50 μM heparin and 5 μM CuCl_2_ and incubated under agitation for 5 days at 37 °C. *A*, negative-staining EM image of copper-oxidized tau fibrils. The scale bar represents 200 nm. *B*, fibrils were sedimented (130,000*g*) and analyzed by nonreducing SDS-PAGE to distinguish between intramolecular and intermolecular disulfide bonds. *C*, a separate badge of fibrils that had not been centrifuged was sonicated and then used as seeds (10%) to assess homotypic (htau40 Ox) and heterotypic (htau40 Red, htau40 SS, htau40 AA, and htau23) recruitment of tau monomers. All reactions were sedimented and analyzed by SDS-PAGE and Coomassie staining (*left panel*). Quantification by gel densitometry (*right panel*). Statistical comparison was performed using paired *t* tests. ∗∗*p* = 0.0066; ∗∗∗*p* ≤ 0.0004. *D*, kinetic analysis of fibril growth. Ten percent copper (II)-oxidized seeds were mixed with variant tau monomers, and fibril growth was monitored by ThT fluorescence. Monomers added were htau40 Ox (*blue trace*); htau40 Red (*red trace*); htau40 SS (*gray trace*); htau40 AA (*green trace*); htau23 (*orange trace*); and seed control (*purple trace*). All ThT measurements were carried out in triplicate. All error bars represent mean ± SD. *E*, SDS-PAGE analysis of htau40 Red and htau23 monomer recruitment onto 10% htau40 SS seeds (lacking cysteines) that were formed in the presence of CuCl_2_ as aforementioned. H_2_O_2_, hydrogen peroxide; n, number of independent replicates; P, pellet; Red, reduced; S, supernatant.

To generate copper-oxidized tau fibrils, htau40 monomers were incubated for 5 days in the presence of heparin and Cu^2+^. Negative-staining TEM analysis revealed that the fibrils had a similar appearance as those formed from H_2_O_2_-oxidized tau (Figs. 2 and 6*A*). When sedimented and analyzed by nonreducing SDS-PAGE, most of the protein was monomeric (Fig. 6*B*), indicating that the disulfide bonds in htau40 were overwhelmingly intramolecular and not intermolecular. Fibrils that had not been centrifuged were then fractured by sonication, and their seeding competency was analyzed. Compact tau monomers (htau40 Ox) were effectively recruited onto these seeds (91%), whereas htau23, htau40 SS, and htau40 AA monomers were not (Fig. 6*C*). Htau40 Red monomers were partially recruited onto the seeds (56%). Similar results were obtained when fibril growth was monitored by ThT fluorescence. Htau40 Ox monomers grew rapidly onto the copper-oxidized fibril seeds, whereas htau40 Red monomers only grew slowly (Fig. 6*D*). The slow growth can be ascribed to the slow oxidation of latter monomers, with oxidation being required for recruitment. Htau40 monomers with cysteine substitutions and htau23 monomers did not grow at all (Fig. 6*D*). To verify that the observed seeding barriers were due to the disulfide links in the fibril and not due to some other structural modification, the original fibrillization reaction (in the presence of Cu^2+^) was repeated with htau40 SS monomers, which lack cysteines. Notably, in this case, both htau40 and htau23 were recruited onto the seeds (Fig. 6*E*). Together, the data highlight that htau40 fibrils formed in the presence of Cu^2+^ exhibit the same seeding properties as fibrils formed from H_2_O_2_-oxidized htau40 (htau40 Ox) and that these fibrils are structurally distinct from fibrils of nonoxidized htau40.

### Oxidized and reduced tau fibrils possess different stabilities

**Figure 7:**
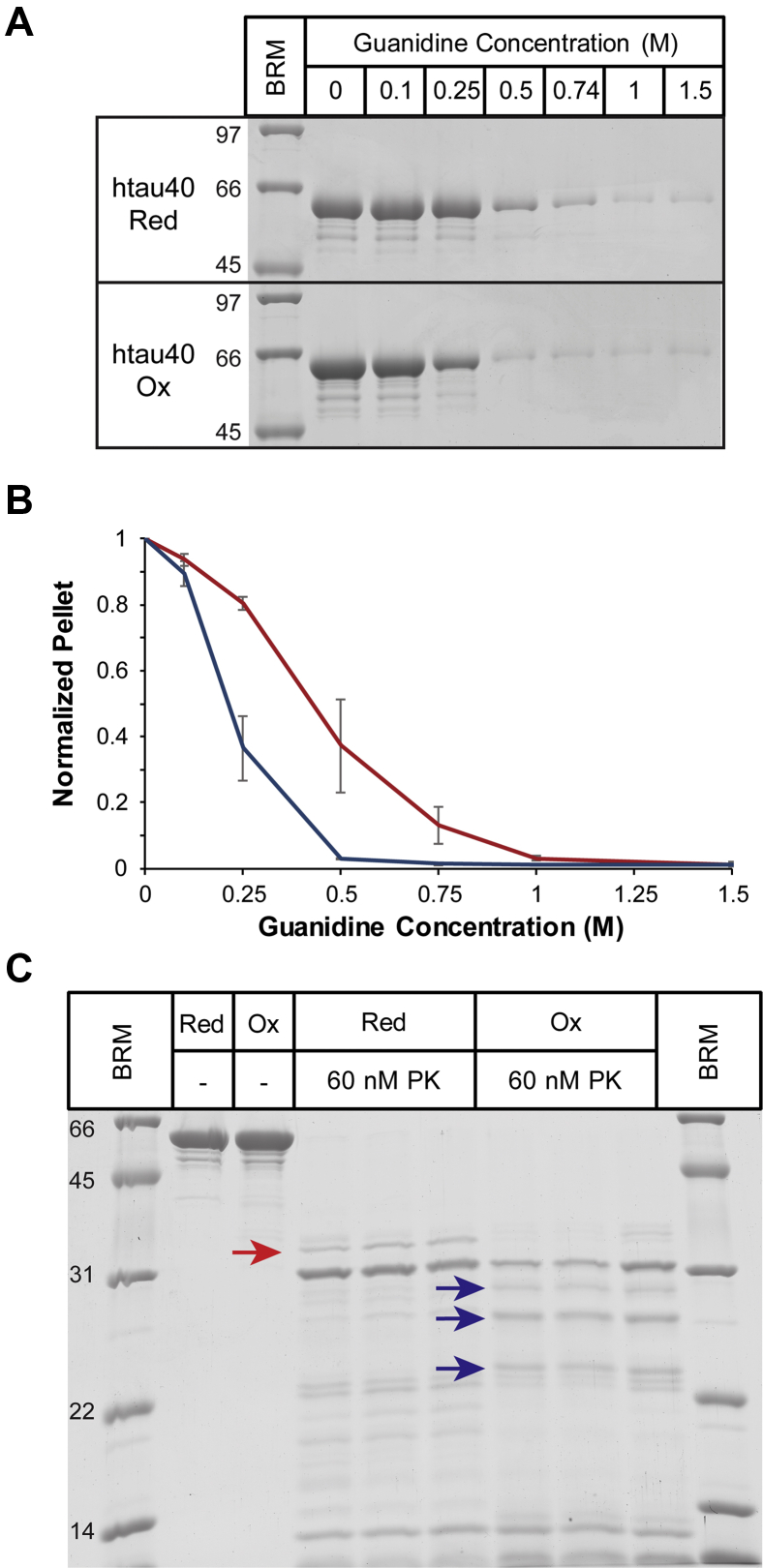
**Htau40 Ox fibrils are less stable than htau40 Red fibrils.***A*, htau40 Ox and htau40 Red fibrils were incubated for 1 h at 22 °C with increasing concentrations of GdnHCl, sedimented at 130,000*g*, and analyzed by SDS-PAGE and Coomassie staining. *B*, quantification of fibril pellets in *A* using gel densitometry. Htau40 Ox fibrils (*blue trace*); htau40 Red (*red trace*). n = 3, mean ± SD. *C*, htau40 Ox and htau40 Red fibrils were incubated for 30 min at 22 °C with 60 nM proteinase K (PK). The reactions were stopped with PMSF and sedimented prior to analysis by SDS-PAGE and Coomassie staining. *Arrows* highlight different proteolytic fragments for htau40 Red fibrils (*red arrow*) and htau40 Ox fibrils (*blue arrows*). The different lanes for the proteolysis reactions represent independent replicates. BRM, broad range marker; GdnHCl, guanidine hydrochloride; Ox, oxidized; Red, reduced.

**Figure 8:**
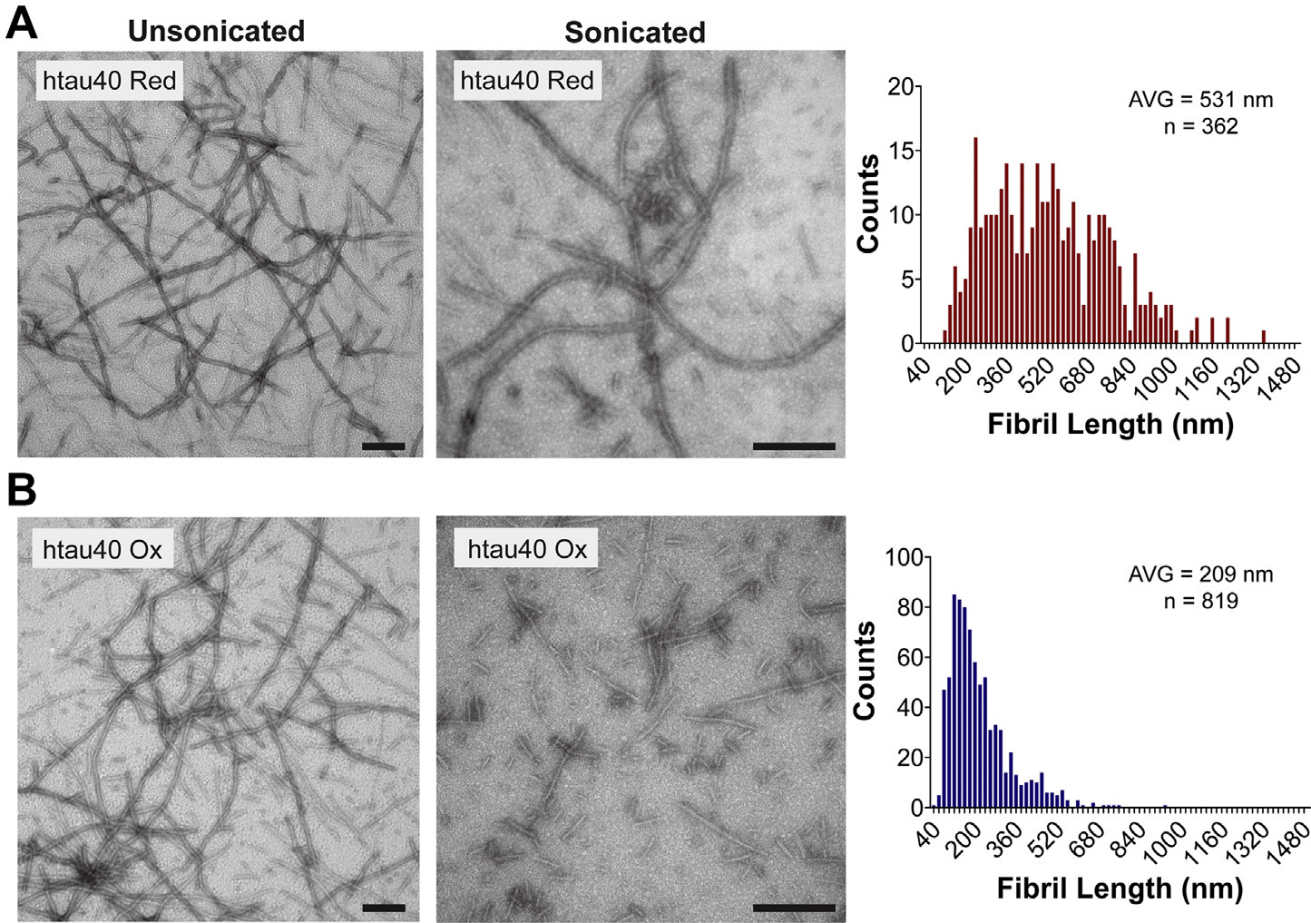
**Htau40 Ox fibrils are more fragile than htau40 Red fibrils.***A* and *B*, fibrils of htau40 Red (*A*) and htau40 Ox (*B*) were imaged by negative-staining EM before and after bath sonication (*left* and *center panels*, respectively). The scale bars represent 200 nm. Length distributions of sonicated fibrils (*right panels*). Ox, oxidized; Red, reduced.

To elucidate structural differences that distinguish oxidized from reduced tau fibrils, we next examined the stabilities of these fibrils. For this purpose, fibrils of htau40 Ox and htau40 Red were first subjected to increasing concentrations of the denaturant guanidine hydrochloride (GdnHCl; Thermo). After a 1-h incubation, the samples were sedimented and analyzed by SDS-PAGE (Fig. 7*A*). Densitometric analysis of the Coomassie-stained bands revealed that htau40 Ox fibrils dissociated at lower denaturant concentrations (Fig. 7*B*, *blue trace*) than htau40 Red fibrils (Fig. 7*B*, *red trace*). To utilize a different readout for stability, the fibrils were next incubated for 30 min with the broad-spectrum serine protease, proteinase K. The banding patterns indicate a larger number of low–molecular-weight fragments for protease-digested htau40 Ox fibrils compared with htau40 Red fibrils (Fig. 7*C*), corroborating that former fibrils are less stable. In a last set of experiments, the mechanical stability of the fibrils was assessed. Specifically, both types of fibrils were subjected for 30 s to bath sonication and then analyzed by negative-staining TEM. Quantification of the electron micrographs reveals that upon sonication, htau40 Ox fibrils exhibit a shorter average length and narrower distribution than htau40 Red fibrils (Fig. 8). These data suggest that htau40 Ox fibrils are more prone to fracture than their reduced counterparts. Collectively, the findings demonstrate that oxidized htau40 fibrils are less stable, both chemically and mechanically, than reduced htau40 fibrils.

### Reducing agents cause disaggregation of htau40 Ox fibrils

**Figure 9:**
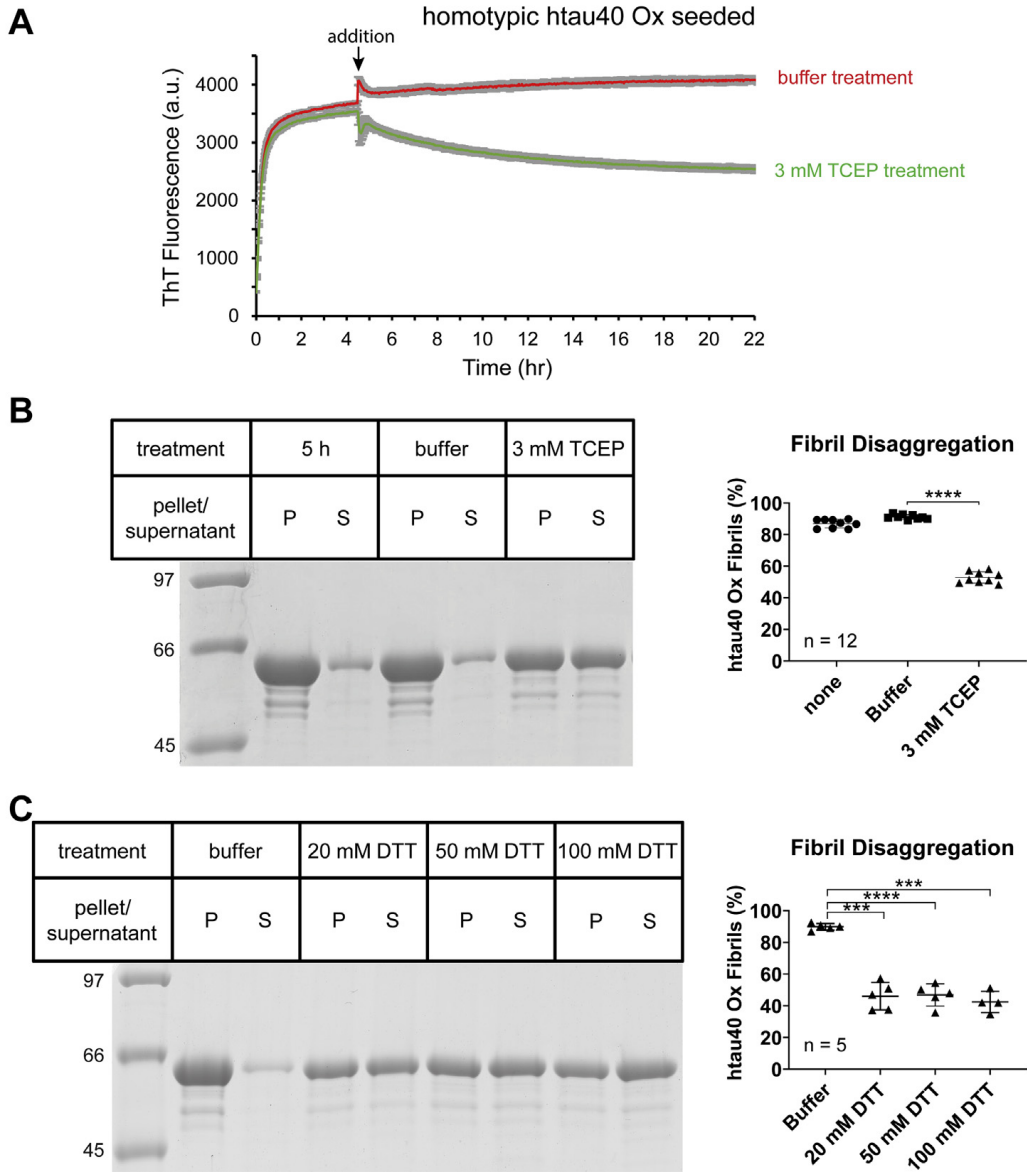
**Reduction of htau40 Ox fibrils results in disaggregation.***A*, 10% htau40 Ox seeds were combined with 10 μM htau40 Ox monomers, and ThT fluorescence was measured at 37 °C over time. After 4.5 h, buffer (*red trace*) or 3 mM TCEP (*green trace*) were added (*arrow*) and the reactions resumed for another 17.5 h. Each reaction was performed in triplicate. Error bars represent mean ± S.D. *B*, the reactions were repeated in the absence of ThT. Addition of buffer or 3 mM TCEP occurred after 5 h. After additional incubation for 16 to 20 h, all reactions were sedimented at 130,000*g*. A separate set of reactions was sedimented after 5 h to verify completion of growth. All samples were analyzed by SDS-PAGE (*left panel*) and quantified densitometrically (*right panel*). *C*, equivalent reactions were carried out in the presence of increasing concentrations of DTT. SDS-PAGE analysis (*left panel*). Quantification (*right panel*). Paired *t* test for statistical comparison: ∗∗∗∗*p* < 0.0001, ∗∗∗*p* ≤ 0.0005. n, number of independent replicates; Ox, oxidized; P, pellet; S, supernatant; TCEP, Tris(2-carboxyethyl)phosphine; ThT, thioflavin T.

Given that the intramolecular disulfide bond is critical for recruitment of htau40 monomers onto oxidized seeds (Figs. 3 and 5), the question arises: What happens when the bond is cleaved after recruitment? Are the fibrils destabilized and disaggregate, or do they stay intact? To address this question, htau40 monomers were mixed and incubated with seeds to allow for fibril formation, and then combined with reducing agent (Tris(2-carboxyethyl)phosphine [TCEP]) to cleave the disulfide bonds. The course of the reaction was monitored by ThT fluorescence. Addition of TCEP during the plateau phase of fibril formation resulted in a 38% decrease in ThT fluorescence compared with buffer control (Fig. 9*A*). These data suggest a reversal in tau fibrillization upon disulfide bond reduction. To obtain additional evidence for this conclusion, the reactions were repeated in the absence of ThT and then centrifuged at 130,000*g*. One set of reactions was sedimented early, at the time of TCEP and buffer treatment, to verify that at that time point fibril formation was complete. Pellets and supernatants of all reactions were analyzed by SDS-PAGE. The results show that tau protein in the two controls (early sedimentation and buffer addition) was found predominantly in the pellets (87% and 91%), whereas tau protein in the TCEP-treated samples was more evenly distributed between pellets (53%) and supernatants (Fig. 9*B*). These data corroborate that the reducing agent causes disaggregation of tau fibrils. Since the process was not complete, otherwise all tau protein would have been found in the supernatant, we next wanted to determine whether the fraction of disaggregated tau could be increased if more stringent reducing conditions were applied. For this purpose, htau40 Ox fibrils were again formed through homotypic seeding, but this time, increasing concentrations of DTT (20, 50, and 100 mM) were applied. Analysis of the sedimented samples revealed similar quantities of proteins in the pellets (46%, 50%, and 45%, respectively) (Fig. 9*C*) indicating that a pool of fibrils remains resistant to these treatments. More importantly, the combined data suggest that a substantial fraction of htau40 Ox fibrils (∼50%) disaggregates upon reduction of the disulfide bond, emphasizing the importance of this bond in stabilizing these fibrils.

### Htau40 Ox fibrils dissociate into tau monomers

**Figure 10:**
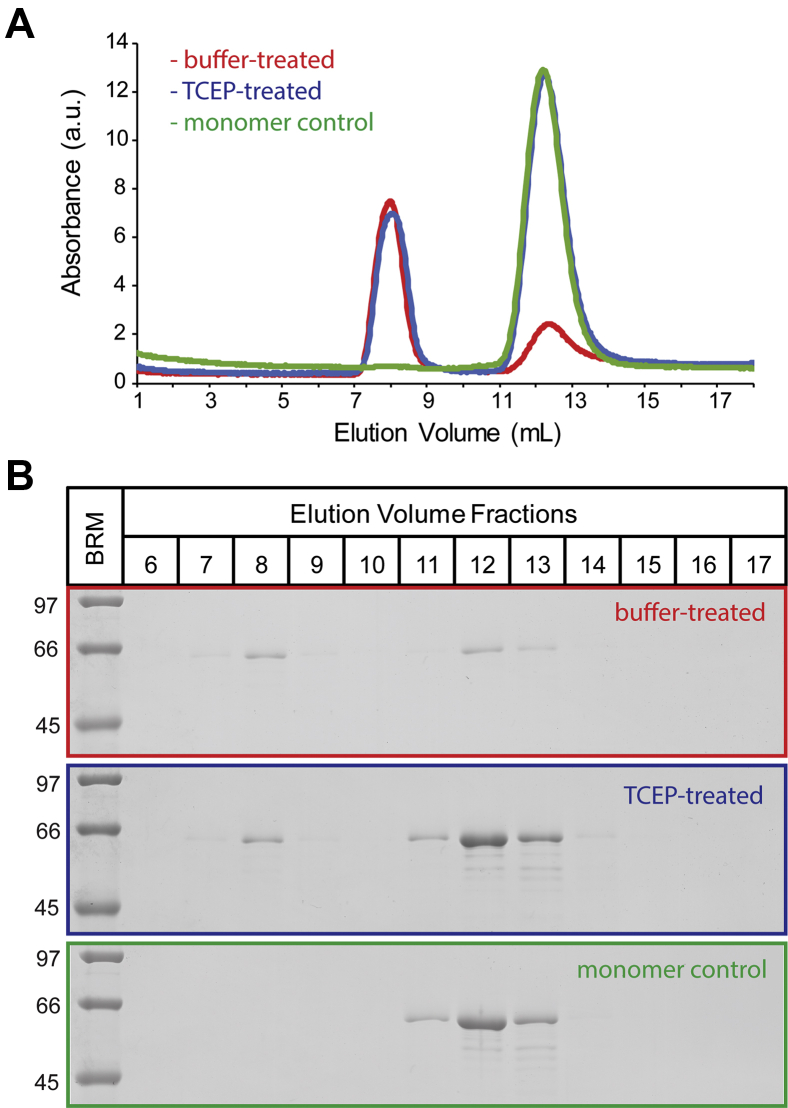
**Htau40 Ox fibrils disaggregate into monomers.** Ten percent htau40 Ox seeds were incubated with 10 μM htau40 Ox monomers for 5 h at 37 °C. Then, buffer or 3 mM TCEP were added, and the incubations were continued for another 16 to 20 h. The samples were sedimented at 130,000*g*. Supernatants were analyzed by size-exclusion chromatography. *A*, elution profiles of supernatants from reactions treated with buffer (*red trace*) or TCEP (*blue trace*). Htau40 monomers loaded as a control (*green trace*). *B*, SDS-PAGE analysis of eluted fractions. Color coding as in (*A*). BRM, broad range marker; Ox, oxidized; TCEP, Tris(2-carboxyethyl)phosphine.

The dissociation of tau fibrils could lead to a broad range of molecular species, including monomers, oligomers, and short fibril fragments. These products are expected to be contained in the supernatant of the centrifuged sample (130,000*g* spin) (43). In a next set of experiments, we set out to analyze this supernatant using size-exclusion chromatography. Fibrils were formed as before using htau40 Ox seeds to initiate aggregation. The fully formed fibrils were then treated with either TCEP or buffer and incubated for 16 to 20 h. After centrifugation, the supernatants were loaded onto a column (Superdex 200) for protein separation. Purified htau40 monomers were loaded as a control. The elution profile of the TCEP-treated samples (Fig. 10*A*, *blue trace*) revealed a major peak at 11 to 13 ml that matched the elution peak of monomeric tau (Fig. 10*A*, *green trace*). A second elution peak at 7 to 8 ml matched the major peak observed in the buffer-treated sample (Fig. 10*A*, *blue trace*). This peak represents tau aggregates that are too large to enter the gel beads but too small to be sedimented. Importantly, the key difference between the elution profiles of buffer-treated and TCEP-treated samples is the largely increased monomer peak observed for latter (Fig. 10*A*). The eluted fractions were next analyzed by SDS-PAGE (Fig. 10*B*). Densitometric analysis revealed that 88% of the total protein in the supernatants of TCEP-treated samples is monomeric (fractions 11–13). Only 12% of the protein appeared in the aggregate fractions at 7 to 8 ml. Importantly, these aggregates are also present in the supernatants of buffer-treated samples (Fig. 10, *A* and *B*), suggesting that they had not been generated upon TCEP treatment. Collectively, the data indicate that htau40 Ox fibrils dissociate into monomers when subjected to reducing conditions.

## Discussion

**Figure 11:**
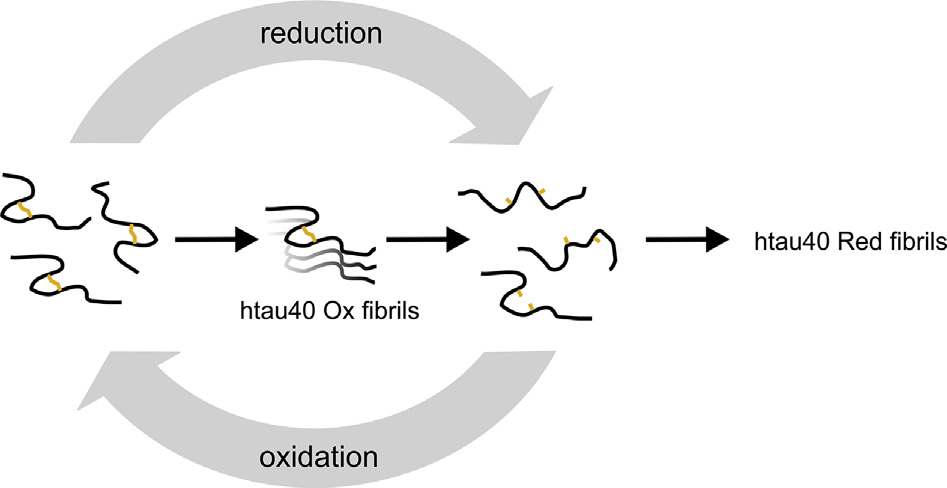
**A binary redox switch in 4R tau controls fibril assembly and disassembly.** Compact and oxidized tau monomers with an intramolecular disulfide bond between cysteines 291 and 322 assemble into stable fibrils (htau40 Ox fibrils). Breakage of the disulfide bond, like the release of a compressed spring, opens the compact structure resulting in fibril disassembly (*center pathway*). The reduced monomers are unable to be recruited back onto oxidized fibrils unless their disulfide bonds are reestablished. Reduced monomers form fibrils on their own. Those fibrils have a different structure from oxidized fibrils and are unable to recruit compact tau monomers. 4R, four-repeat.

Oxidative stress is a contributing factor to aging and has been linked to AD and other neurodegenerative disorders (46, 47, 48). Intermolecular disulfide linkages between tau proteins have been associated with enhanced aggregation (28), whereas the implications of intramolecular disulfide linkages, occurring in only 4R tau, are still controversial (28, 34, 35, 39). Given the central role of tau in disease, understanding the molecular characteristics of all its different species is essential. In this study, we set out to determine the aggregation properties of compact htau40 monomers with intramolecular disulfide bonds. We observed that the monomers form fibrils that are highly efficient in homotypic seeding, that is, under the given conditions, compact tau monomers are prone to aggregation. The oxidized fibrils are different from fibrils formed under reducing conditions in that they are more fragile and less stable toward proteolysis and denaturation. These observations are consistent with the work published by Furukawa *et al.* (35), which described a smaller core structure for fibrils formed from compact tau. Unique to the current study is the identification of very strong seeding barriers that prevent recruitment of reduced tau monomers onto oxidized seeds. These barriers also hold when htau40 monomers with native cysteines replaced by serines or alanines are offered to the seeds or when htau23 monomers with only a single cysteine are employed, suggesting that the compact restrained structure of oxidized htau40 monomers is required for recruitment. Even more remarkable, fibrils composed of oxidized htau40 disaggregate into monomers when exposed to reductants. The intramolecular disulfide bond in htau40 thus serves as a binary redox switch that controls the aggregation and disaggregation of these fibrils (Fig. 11). But what is the energetic basis for this?

The formation of a disulfide bond in htau40 reduces the conformational entropy of the disordered monomer. It is possible that this reduction in entropy could be a driving force for the recruitment of compact tau monomers into oxidized fibrils as the entropy change for assembly becomes less negative. In the case of reduced tau monomers and their mimics, no such stabilization occurs, providing a plausible explanation for the observed seeding barrier and the disaggregation of oxidized fibrils upon reduction. Conformational entropy has long played a role in rationalizing the stabilization of proteins with disulfide bonds (49, 50, 51, 52), although it is now recognized that solvent enthalpy and other effects can contribute in sometimes significant ways (53, 54). One difference between globular proteins and tau is that tau folds on a template (7) and not in isolation. Consequently, interactions between the outer fibril layer and the incoming monomer will factor into the overall stability of the fold. The aggregation of tau also depends on the presence of negatively charged cofactors, which have been found to bind both, monomers (55, 56, 57) and fibrils (20, 21, 22). Varied interactions with heparin, conformational imprinting, or structural rearrangements could be some reasons why the fibrils investigated in the current study do not fully dissociate upon reduction. Intramolecular disulfide bonds are observed in the fibril polymorphs of various amyloidogenic proteins, including β2-microglobulin (58), immunoglobulin light chain (59, 60, 61), and human prion protein (62, 63). However, there is no evidence that the reduction of disulfide bonds in these fibrils results in disaggregation. The observed reversibility in tau aggregation hinges on the exquisite energetics, favoring disordered monomers in the reduced state and ordered fibrils in the oxidized state. To our knowledge, such redox-controlled switch has not yet been described for any other amyloid fibril.

Reversibility in fibril formation is typically associated with functional amyloids, where changes in protein modification, pH, concentration, or buffer can cause a switch in the assembly status (64, 65). One example is the disaggregation of assembled peptide hormones after release from secretory vesicles (66, 67). Tau protein is predominantly located in the reducing environment of the cytosol, but situations of oxidative stress increase the reduction potential. An array of antioxidant enzymes has evolved to protect against oxidative assaults in the cell and ensure redox homeostasis (68). Tau protein that is secreted into extracellular space (69) occupies an oxidizing environment (70). The transport of tau between intracellular and extracellular space thus constitutes a shuttling between different redox environments; and it is likely that this will affect the oxidation status of the cysteines. It must be stressed that none of the pathological fibrils containing 4R tau for which high-resolution structures are available show disulfide bonds (30, 32, 33). The cysteines at positions 291 and 322 in these fibrils are not in close proximity. Therefore, it is currently unknown whether oxidized tau fibrils could play a pathological role. However, to date, only a fraction of the vast spectrum of fibrils found in tauopathies has been elucidated to atomic or near-atomic resolution. One thing is clear, tau monomers with intramolecular disulfide bonds are incompatible to grow onto reduced fibrils and vice versa. Segregation of 4R tau into separate pools would lower the monomer concentration available for aggregation and could thereby be protective. Although the full scope of biological consequences requires further study, the herein identified redox switch in 4R tau establishes a powerful means for controlling its structure and function. The findings should reinvigorate interest in better understanding the role of oxidized tau in the human brain.

## Experimental procedures

### Constructs

The cloning of htau40 and htau23 into pET-28 using the Nco1 and Xho1 restriction sites was previously described (37). Cysteine-free constructs of htau40 were generated by replacing the two native cysteines with serines (htau40 SS) or alanines (htau40 AA) following the QuikChange site-directed mutagenesis method (Agilent Technologies). The correctness of all sequences was confirmed by DNA sequencing.

### Expression and purification

All proteins were expressed and purified according to published protocols (37, 71). In brief, BL21 (DE3)-competent *Escherichia coli* cells were transformed by heat shock with the desired pET-28 plasmids and then grown on LB (Miller) agar plates. Single colonies were transferred into LB medium and grown under agitation for 15 to 17 h at 37 °C. Next, the cultures were diluted 1:100 with LB medium and again grown under agitation at 37 °C until the absorbance at 600 nm reached 0.7 to 1. Selection occurred in the presence of kanamycin (50 μg/ml in plates and 20 μg/ml in solution). Protein expression was induced with the addition of 0.5 mM isopropyl β-D-1-thiogalactopyranoside (Gold Biotechnology). Cultures were incubated for another 3.5 h at 37 °C before being sedimented and resuspended in buffer (500 mM NaCl and 20 mM Pipes [pH 6.5]; Research Products International), 1 mM EDTA (Fisher Scientific), and 50 mM β-mercaptoethanol (Fisher BioReagents). Cells were then heated for 20 min at 80 °C and tip sonicated for 1 min on ice. To separate soluble protein from cellular debris, the samples were centrifuged for 30 min at 15,000*g*. Soluble tau was precipitated through gentle agitation with 55 to 60% w/v ammonium sulfate for 15 to 20 h at 22 °C and sedimented for 20 min at 20,000*g*. Tau pellets were resuspended in water with 2 mM DTT (Gold Biotechnology), tip sonicated for 2 to 3 min, syringe filtered (GxF/GHP 0.45 μm), and loaded onto a cation exchange column (mono S 10/100 GL; GE). A linear NaCl gradient (50–1000 mM NaCl with 20 mM Pipes [pH 6.5], 2 mM EDTA, and 2 mM DTT) was used to elute proteins, and fractions were assessed by SDS-PAGE using SDS sample buffer (62.5 mM Tris at pH 6.5 [Sigma]; 4% SDS (J.T. Baker); 10% sucrose [MP Biomedicals]; 5% 2-mercaptoethanol [Fisher Scientific], 1.5 mM bromophenol blue [Sigma]). Fractions containing the highest concentration of tau were pooled and loaded onto a Superdex 200 size exclusion column, then eluted with buffer containing 100 mM NaCl, 20 mM Tris at pH 7.4, 1 mM EDTA, and 2 mM DTT. Protein purity was assessed by SDS-PAGE, and samples were pooled accordingly. Protein precipitation was achieved by mixing the pooled fractions with an equimolar volume of methanol and incubating them on ice for 12 to 20 h. The precipitated protein was then pelleted by centrifugation for 10 min at 15,000*g*, washed with methanol containing 2 mM DTT, and stored at −80 °C until further use.

### Protein solubilization and oxidation of htau40

Protein pellets were resuspended in 8 M GdnHCl. The samples were then loaded onto PD-10 columns (GE Healthcare) and eluted with “reaction buffer” (100 mM NaCl, 40 mM Hepes [J.T. Baker], and 0.1 mM NaN_3_ [Fisher Scientific] at pH 7.4). Protein concentration was determined using the bicinchoninic acid (BCA) assay (Pierce). Samples were stored on ice in the presence or the absence of 0.5 mM TCEP. TCEP was added to those proteins in which cysteines had to be preserved in their reduced states (htau23 and htau40 Red). Htau40 Ox was generated by adding 5 mM H_2_O_2_ (Sigma–Aldrich 30% [w/w] in water) to 20 μM htau40 (no TCEP) and incubating the mixture for 15 h at 22 °C. The reactions were then dialyzed at 5 °C (dialysis tubing, spectra/Por flat width 10 mm, and molecular weight cutoff of 12–14 kDa) and further purified by size-exclusion chromatography (GE Healthcare Superdex 200 10/300 GL) using reaction buffer for protein elution. The fractions were analyzed by nonreducing SDS-PAGE to differentiate between proteins with intermolecular *versus* intramolecular disulfide bonds. Latter proteins, which appeared as monomers in the gel, were pooled, and protein concentrations were determined using the BCA assay. The same oxidation/purification procedure was repeated with htau40 AA and htau40 SS monomers, generating tau controls, referred to as htau40 AA Ox and htau40 SS Ox.

### CW EPR sample preparation and measurement

CW EPR spectroscopy was used to confirm that the cysteines in htau40 Ox monomers were inaccessible for thiol crosslinking. For this purpose, purified protein was taken up in 8 M GdnHCl and incubated for 1 h at 22 °C with a 10-fold molar excess of the paramagnetic spin label MTSL (Toronto Research Chemicals taken up in dimethyl sulfoxide to 40 mg/ml). Excess label and denaturant were removed by gel filtration using PD-10 columns. The labeling procedure was repeated with nonoxidized htau40 monomers and oxidized monomers (htau40 Ox) that had been incubated with 20 mM DTT for 5 h (to reduce the disulfide bonds) and then dialyzed (to remove DTT). Both samples were expected to have free cysteines available for labeling and thus served as controls. CW EPR spectra (20 scans) of samples containing 5 μM monomers were collected at X-band (9.5 GHz) at 22 °C with 150 G sweep width, 12.05 mW power, and 1 G modulation amplitude on a Bruker EMX Plus spectrometer fitted with an ER 4119HS resonator.

### Native PAGE

The protocol for native gel electrophoresis was an adaptation of previously described methods (39, 72). The electrophoretic system was comprised of an upper 4% and a lower 8% acrylamide gel (37.5:1 acrylamide/bisacrylamide) containing buffer (30 mM β-alanine [Sigma] and 20 mM lactic acid [Sigma]) that was adjusted to pH 4.4 and 3.8, respectively. The buffers were identical to those used in the respective upper and lower gel compartments of the Mini-Protean III cell (Bio-Rad). Three types of htau40 monomers at 5 μM concentration were employed: reduced, oxidized, and oxidized incubated for 1 h with 20 mM DTT. The proteins were mixed with 2.5× sample buffer (0.01% methyl green [Sigma], 25% glycerol, 75 mM β-alanine, and 55 mM lactic acid) and then loaded onto the gel. The gel was run for 2 h at 250 V with polarity reversed and then stained with Coomassie blue for analysis.

### Seed preparation

Htau40 Ox fibrils were formed by incubating 500 μl of 25 μM hatu40 Ox monomers and 50 μM heparin (Celsus; average molecular weight of 4400 Da) in reaction buffer stirring with a teflon-coated micro stir bar (5 × 2 mm) at 160 rpm for 5 to 7 days at 37 °C. Htau40 Red fibrils were formed the same way, except that htau40 Red monomers were used and that 0.5 mM TCEP was included during the incubation. A separate set of reactions with htau40 (not treated with TCEP) and htau40 SS monomers also contained 5 μM CuCl_2_. To generate short fibril fragments (seeds) after multiple days of agitated incubation, fibrils (500 μl) were subjected to 30 s of sonication on ice at 20% power with a Fisher Scientific sonifier (model 100 with a 2 mm tip).

### Seeded reactions

All reactions referred to as seeded reactions included the following components: 10% seeds (monomer equivalents), 10 μM tau monomer in reaction buffer, and 20 μM heparin. Unless noted otherwise, fibrils were generated by quiescent incubation for 20 to 24 h at 37 °C. The fibrils were then sedimented by centrifugation for 30 min at 10 °C and 130,000*g* (Beckman L7-55 ultracentrifuge).

Pellets and supernatants were separated, and volumes were adjusted with SDS sample buffer so that equivalent amounts could be analyzed by 12% SDS-PAGE and Coomassie staining. In cases where it was important to differentiate between intramolecular and intermolecular disulfide bonds in tau, reducing agent was absent in the sample buffer. Fibrillization was quantified using ImageJ (National Institutes of Health) and GraphPad Prism 7 software (GraphPad Software Inc). The percentage of growth was determined by dividing the band intensity of the pellet by the total band intensity (pellet plus supernatant) and multiplying by 100. Each data point in the scatter plots represents an independent replicate (obtained from different seed batches).

### TEM

Formvar/carbon-coated 200 mesh copper grids (Electron Microscopy Sciences) were covered for 1.5 min with 10-μl droplets of diluted tau fibrils (∼2.5 μM), blotted on filter paper to remove excess liquid, covered for 1.5 min with droplets of 2% uranyl acetate (Electron Microscopy Sciences; 0.2 μm syringe filtered), and again blotted on filter paper. The grids were air dried and then stored until use. Images were recorded with an FEI Tecnai T12 Biotwin electron microscope at 100 KeV equipped with a Gatan CCD camera. Fibril lengths were quantified using ImageJ software.

### Fibril elongation

To assess the kinetics of fibril elongation, 10 μM tau monomers were mixed with 20 μM heparin, 5 μM ThT, and 10% seeds (monomer equivalents). The mixtures were incubated quiescently at 37 °C in a BGM Labtech FLUOstar Omega plate reader while monitoring the fluorescence emission in real time at 480 nm. The excitation wavelength was set at 440 nm. All readings were taken through the bottom of a 96-well optical PolymerBase plate (Thermo Scientific).

### Bismaleimide crosslinking of htau40 monomer

A methanol-precipitated pellet of htau40 was taken up in 8 M GdnHCl, mixed with a threefold molar excess of bis(maleimido)ethane (Thermo Scientific; taken up in dimethyl sulfoxide to 20 mM), and quiescently incubated for 1 h at 22 °C to allow for crosslinking of the native cysteines. The reaction was quenched with 500 mM DTT and then applied onto a PD-10 column. Eluted protein was loaded on a size-exclusion column (GE Healthcare Superdex 200 10/300 GL) to separate tau monomers with intramolecular crosslinks from higher order species. Monomer fractions were pooled based on SDS-PAGE analysis, and concentration was determined using the BCA assay.

### Aerobic and anaerobic reactions

The experiments explicitly described as anaerobic were carried out in a Coy Lab Products anaerobic chamber and glove box. Oxygen content was kept between 10 and 20 parts per million using a Coy Anaerobic Monitor (CAM-12 detector) and compressed gas (nitrogen and hydrogen). Buffer to be used was purged with compressed nitrogen gas for 30 min prior to entering the chamber. After dissolving a methanol-precipitated pellet of htau40 in ambient room conditions with GdnHCl, the dissolved protein was taken into the chamber where it was run through a PD-10 column in the same manner as described previously. Following elution off the column, protein concentration was determined by the BCA assay. Fibril formation was initiated by mixing 10 μM htau40 monomer with 20 μM heparin and 10% seeds (monomer equivalents). Reactions were incubated quiescently for 20 to 24 h at 37 °C in a digital dry bath inside the chamber with tubes closed (anaerobic) or outside the chamber on the bench at 22 °C with tubes open (aerobic). All samples were centrifuged for 30 min at 130,000*g* as before, followed by SDS-PAGE analysis.

### GdnHCl denaturation

Fibrils of htau40 Ox and htau40 Red (formed as described under seeded reactions) were aliquoted and mixed with increasing concentrations of GdnHCl (0–1.5 M) and incubated for 1 h at 22 °C. The samples were then sedimented at 130,000*g* to separate fibrils from denatured monomers, and pellets were analyzed by SDS-PAGE and Coomassie staining. Band intensities were quantified using ImageJ software.

### Limited proteolysis

Fibrils of htau40 Ox and htau40 Red (formed as described under seeded reactions) were mixed with 60 nM proteinase K (Promega) or buffer (controls) and incubated for 30 min at 22 °C. Proteolysis was stopped by addition of 4 mM phenylmethylsulfonyl fluoride (Sigma). The samples were then analyzed by SDS-PAGE (15%) and Coomassie staining.

### Fibril fragility

To determine differences in fibril fragility 100 μl of htau40 Ox and htau40 Red fibrils (generated through homotypic seeding) were sonicated for 30 s in a Qsonica bath sonicator (Q700 connected to a 5.5‘’ cup horn) at 5% power. The samples were then analyzed by negative-staining TEM.

### Dissociation of htau40 Ox fibrils by reducing agents

The dissociation of htau40 Ox fibrils upon addition of reducing agents was monitored by ThT fluorescence and sedimentation. Homotypically seeded reactions were set up as described previously. Specifically, 10 μM htau40 Ox monomers in reaction buffer were mixed with 20 μM heparin, 5 μM ThT, and 10% htau40 Ox seeds (monomer equivalents) and incubated for 22 h at 37 °C. Fluorescence emission was continuously monitored at 480 nm with the excitation wavelength set at 440 nm. At 4.5 h (upon reaching the plateau phase), either 3 mM TCEP or buffer (control) was added to the reactions. Dissociation was assessed by the difference in fluorescence intensities at the end of incubation. The reactions were repeated in the absence of ThT. In this case, aggregation was allowed to proceed for 5 h before a subset of the reactions was sedimented for 30 min at 130,000*g* to confirm completion of fibril formation. The remaining reactions were combined with reducing agents (3 mM TCEP or 20–100 mM DTT) and incubated for a further 16 to 20 h at 37 °C. These reactions were then sedimented as well. Pellets and supernatants were taken up in sample buffer, analyzed by SDS-PAGE and Coomassie staining, and quantified. To assess the oligomerization status of tau, the supernatants from the TCEP-treated and buffer-treated reactions were fractionated by size-exclusion chromatography (GE Healthcare Superdex 200 10/300 GL), and the elution profiles were compared with an htau40 monomer control (5 μM). The eluted fractions were further analyzed by SDS-PAGE and Coomassie staining.

## Data availability

All the data are contained within the article.

## Supporting information

This article contains supporting information.

## Conflict of interest

The authors declare that they have no conflicts of interest with the contents of this article.
