# Supplementary material for: A thiol-based intramolecular redox switch in four-repeat tau controls fibril assembly and disassembly
Source: J Biol Chem. 2021 Jul 31;297(3):101021. doi: 10.1016/j.jbc.2021.101021 (PMC8387771; doi:10.1016/j.jbc.2021.101021)
Supplement: Supplemental Figures S1–S3 [file mmc1.pdf]

SUPPORTING INFORMATION

A thiol-based intramolecular redox switch in four-repeat tau controls fibril assembly and disassembly

Hilary A. Weismiller, Tyler J. Holub, Brad J. Krzesinski, and Martin Margittai*

From the Department of Chemistry and Biochemistry, University of Denver, Denver, CO 80208, USA

Running title: Thiol-based redox switch in four-repeat tau

*To whom correspondence should be addressed: Martin Margittai, Department of Chemistry and Biochemistry, University of Denver, 2190 East Iliff Ave, Denver CO 80208. Tel: (303)-871-4135; Fax: (303)-871-2254. E-mail: martin.margittai@du.edu

Contents

Figure S1. Htau40 Ox monomers do not aggregate under quiescent conditions.

Figure S2. Htau40 AA and htau40 SS monomers that are treated with hydrogen peroxide do not grow onto htau40 Ox seeds.

Figure S3. Htau40 monomers with an intramolecular bismaleimide crosslink are not recruited onto htau40 Ox seeds.

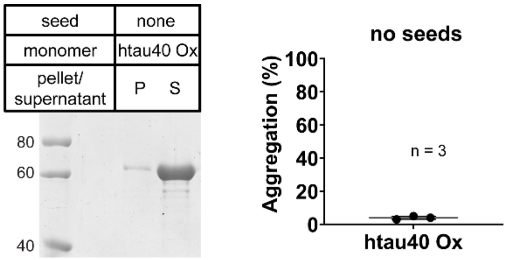

Figure S1. Htau40 Ox monomers do not aggregate under quiescent conditions. To test for spontaneous nucleation, 10 µM htau40 Ox monomer was mixed with 20 µM heparin, incubated quiescently at 37 °C for 24 h and then sedimented at 130,000 x g. Equivalent volumes of pellet and supernatant were subjected to SDS-PAGE and visualized by Coomassie staining (left panel). Quantitative analysis (right panel) was based on gel densitometry. P, pellet; S, supernatant; n, number of independent replicates. Error bars represent means ±S.D.

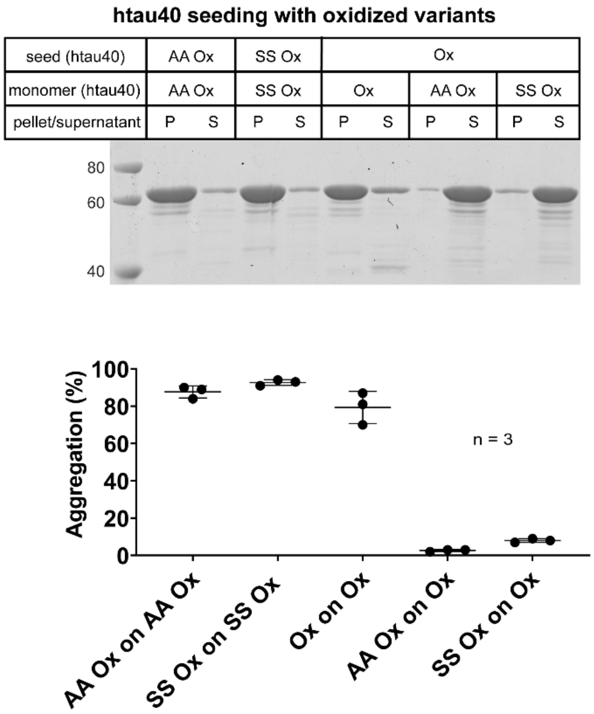

Figure S2. Htau40 AA and htau40 SS monomers that are treated with hydrogen peroxide do not grow onto htau40 Ox seeds. Htau40 AA and htau40 SS monomers (20 μM) were oxidized for 15 h at 22 °C with hydrogen peroxide (5 mM), subjected to size exclusion chromatography (Superdex 200), and then pooled. These monomers are referred to as htau40 AA Ox and htau40 SS Ox. Fibrils were formed under agitation as described for htau40 Ox (Fig. 2), followed by sonication. To assess homo- and heterotypic growth, 10 μM tau monomer was mixed with 10% seed (monomer equivalents) and 20 μM heparin, incubated quiescently at 37 °C for 24 h, and then sedimented at 130,000 x g. Equivalent volumes of pellet and supernatant were subjected to SDS-PAGE and visualized by Coomassie staining (upper panel). The bands were quantified by gel densitometry (lower panel). P, pellet; S, supernatant; n, number of independent replicates. Error bars represent means ±S.D.

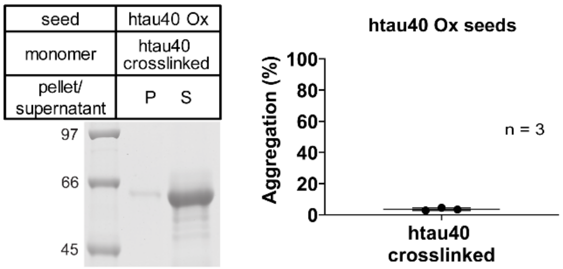

Figure S3. Htau40 monomers with an intramolecular bismaleimide crosslink are not recruited onto htau40 Ox seeds. Htau40 monomers in which the two native cysteines were crosslinked with an 8 Å bismaleimide spacer arm were isolated by size exclusion chromatography. These monomers (10 µM) were then mixed with htau40 Ox seeds (10 %) and incubated for 24 h at 37 °C. After sedimentation, equivalent volumes of pellet (P) and supernatant (S) were analyzed by SDS-PAGE (left panel) and quantified by densitometry (right panel). n, number of independent replicates.
